# Supplementary material for: Skull-Base Chondrosarcoma: A Systematic Review of the Role of Postoperative Radiotherapy
Source: Cancers (Basel). 2024 Feb 21;16(5):856. doi: 10.3390/cancers16050856 (PMC10931115; doi:10.3390/cancers16050856)
Supplement: Supplementary file 1 [file cancers-16-00856-s001.zip › cancers-2787880-supplementary.pdf]

Supplementary

Table S1: Risk of bias assessment of included cohort studies as per ROBINS-I tool [9].

Study	Bias due to confounding	Bias in selection of participants into the study	Bias in classification of interventions	Bias due to deviations from the intended intervention	Bias due to missing data	Bias in measurement of outcomes	Bias in selection of the reported result	Overall risk of bias
**Hug et al., 1999**	Moderate risk	Low risk	Low risk	Low risk	Low risk	Moderate risk	Low risk	Moderate risk
**Rosenberg et al., 1999**	Critical risk	Moderate risk	Low risk	Low risk	No information	No information	Serious risk	Critical risk
**Noel et al., 2001**	Moderate risk	Low risk	Low risk	Low risk	Low risk	Low-moderate risk	Low risk	Moderate risk
**Tzortzidis et al., 2006**	Assessed via JBL checklist	Assessed via JBL checklist	Assessed via JBL checklist	Assessed via JBL checklist	Assessed via JBL checklist	Assessed via JBL checklist	Assessed via JBL checklist	Assessed via JBL checklist
**Schulz-Ertner et al., 2007**	Critical risk	Low risk	Low risk	Low risk	Moderate-serious risk	Low risk	Low risk	Critical risk
**Samii et al., 2008**	Critical risk	Low risk	Low risk	Low risk	Serious-critical risk	Low risk	Low risk	Critical risk
**Sahgal et al., 2015**	Serious risk	Moderate risk	Low risk	Low risk	Low risk	Low risk	Low risk	Serious risk
**Uhl et al., 2014**	Moderate risk	Low risk	Moderate risk	Low risk	No information	Low-moderate risk	Low risk	Moderate risk
**Feuvret et al., 2015**	Moderate risk	Low risk	Moderate risk	Low risk	No information	Low risk	Low risk	Moderate risk
**Weber et al., 2016**	Low risk	High risk	Not applicable	Not applicable	Serious risk	Low risk	Low risk	Moderate risk
**Weber et al., 2016**	Moderate risk	Low risk	Low risk	Low risk	No information/low risk	Low risk	Low risk	Moderate risk
**Vaz-Guimares et al., 2017**	Moderate risk	Low - moderate risk	Low risk	Low risk	Low-moderate risk	Low risk	Low-moderate risk	Moderate risk
**Hasegawa et al., 2018**	Critical risk	Low risk	Low risk	Low risk	No information	Low risk	Low risk	Critical risk
**Simon et al., 2018**	Moderate	Low-moderate risk	Moderate risk	Low risk	Moderate risk	Low risk	Low risk	Moderate risk
**Mattke et al., 2018**	Moderate risk	Low risk	Low risk	Serious risk	Moderate risk	Low risk	Low risk	Moderate risk
**Weber et al., 2018**	Moderate risk	Low risk	Moderate-serious risk	Low risk	Moderate risk	Low-moderate risk	Low risk	Moderate-Serious risk
**Holtzman et al., 2019**	Critical risk	Low risk	Low risk	Low risk	No information	Low risk	Low risk	Critical risk
**Hasegawa et al., 2021**	Moderate risk	Moderate risk	Low risk	Low risk	No information	Low risk	Low risk	Moderate risk
**Kawashima et al.,2022**	Serious risk	Low risk	Low risk	Low risk	No information	Low risk	Low risk	Serious bias
**Pattankar et al., 2022**	Critical risk	Low risk	Low risk	Low risk	No information	Low risk	Low risk	Critical risk
**Al Shaibibi et al., 2006**	Assessed via JBL checklist	Assessed via JBL checklist	Assessed via JBL checklist	Assessed via JBL checklist	Assessed via JBL checklist	Assessed via JBL checklist	Assessed via JBL checklist	Assessed via JBL checklist
**Liu et al., 2023**	Moderate risk	Low risk	Low risk	Low risk	No information	Low risk	Low risk	Moderate risk

Table S2: Risk of bias assessment of included case series assessed as per JBI Checklist for case series [10].

Study	Inclusion criteria	Condition measured	Identification of disease	Consecutive inclusion	Complete inclusion	Reporting demographics	Reporting clinical information	Outcomes/ follow up	Setting	Statistical analysis
**Tzortzidis et al, 2006**	Yes	Yes	Yes	Yes	Yes	No	Yes	Yes	No	Yes
**Al Shaibibi et al., 2023**	Yes	Yes	Yes	Yes	Yes	Yes	Yes	Yes	No	Yes
